# The Emerging Mycotoxin 2-Amino-14, 16-Dimethyloctadecan-3-ol (AOD) Alters Transcriptional Regulation and Sphingolipid Metabolism and Undergoes *N*-Acylation by HepG2 Cells

**DOI:** 10.3390/toxins17080413

**Published:** 2025-08-15

**Authors:** Shenlong Mo, Zhenying Hu, Huaiyi Zhu, Boming Yu, Xiaoyan Chen, Yu Chen, Alfred H. Merrill, Jingjing Duan

**Affiliations:** 1 Jiangxi Key Laboratory of Aging and Disease, Sphingolipid Metabolism and Aging, Human Aging Research Institute (HARI) and School of Life Science, Nanchang University, Nanchang 330031, China; shenlong_mo@163.com (S.M.); whozing@ncu.edu.cn (Z.H.); huaiyi_zhu@email.ncu.edu.cn (H.Z.); bomingyu666@163.com (B.Y.); xyanchen9614@163.com (X.C.); ncuskchenyu2020@163.com (Y.C.); 2 School of Biological Sciences, the Parker H. Petit Institute for Bioengineering and Bioscience, Georgia Institute of Technology, Atlanta, GA 30332, USA

**Keywords:** mycotoxin, sphingolipids, 1-deoxysphingolipid, AOD, ceramide synthase, stress-responsive signaling

## Abstract

2-Amino-14,16-dimethyloctadecan-3-ol (AOD) is commonly found in foods contaminated with *Fusarium avenaceum*, particularly cereals or fruits, and is structurally related to *Fusarium* mycotoxins (fumonisins) and mammalian sphingoid bases, especially 1-deoxysphinganine (m18:0); therefore, it might enter systemic circulation and tissues upon dietary intake. Knowledge about what happens when cells are exposed to AOD is limited, but it has been reported to be cytotoxic and to induce vacuolization in HepG2 cells. We also found that AOD is cytotoxic for HepG2 cells, but even at a concentration where cell viability remained above 85% (5 μM), it altered 24 differentially expressed genes based on RNA sequencing-based transcriptomic profiling. Among these genes, 13 were shared with cells treated with m18:0. These overlapping differentially expressed genes were primarily enriched in activated stress response pathways of cells, including the upregulation of specific genes in the hypoxia-inducible factor 1α (HIF-1α) signaling pathway, such as hexokinase 1 (*HK1*) and egl-9 family hypoxia-inducible factor 3 (*EGLN3*), the activation of key components in the p53 signaling pathway, and the induction of cellular senescence-associated transcriptional programs involving serpin family E member 1 (*SERPINE1*). Transcriptional analysis of genes related to sphingolipid metabolism showed that treatment with AOD increased the mRNA expression of ceramide synthase 4 (*CerS4*), sphingosine-1-phosphate phosphatase 1 (*SGPP1*), and UDP-glucosylceramide glucosyltransferase (*UGCG*), while decreasing the expression of dihydroceramide desaturase 1 (*DEGS1*) and fatty acid desaturase 3 (*FADS3*), a pattern of gene expression changes that mirrored the alterations observed with m18:0 treatment. Lipidomic analyses revealed that AOD significantly perturbed the sphingolipid composition of HepG2 cells, specifically increasing hexosylceramide content while decreasing ceramide and sphingomyelin levels. Moreover, AOD was found to undergo intracellular metabolism to *N*-acyl-AODs, perhaps by ceramide synthase(s), since this acylation was inhibited by fumonisin B1 (FB1). These findings demonstrate that AOD or possibly its *N*-acyl metabolites can alter cellular sphingolipid metabolism and affect the expression of genes involved in cell stress. These new insights call for more studies of the impact of this food contaminant on cells and the implications for human health.

## 1. Introduction

2-Amino-14,16-dimethyloctadecan-3-ol (AOD) is a sphingoid base analog that accumulates in cereals and fruits contaminated by *Fusarium avenaceum* [1,2,3,4,5]. For example, Uhlig et al. detected AOD in 35% of field grain samples with concentrations reaching 10.8 mg/kg [5]. AOD is structurally similar to the well-known *Fusarium* mycotoxins (fumonisins) [6,7] and, in addition, resembles 1-deoxysphinganine (m18:0, Figure 1A), which is a cytotoxic “atypical” 1-deoxysphingoid base (i.e., lacking the hydroxyl group at the C1 position found in typical sphingolipids) [8,9]. This structural feature of atypical 1-deoxysphingolipids prevents their metabolism to “canonical” sphingolipids such as sphingomyelins, glycosylceramides, and sphingoid base-1-phosphates [8,9], and instead, they disrupt cell regulatory systems that can result in hepatotoxicity and retinal toxicity [10,11,12,13] and sensory and autonomic neuropathies [14,15,16,17]. Given the structural similarities between AOD, fumonisins, and m18:0, one would predict that this fungal metabolite might have at least some overlapping cellular effects, such as shared impacts on key cellular processes like metabolism and stress responses.

AOD has been found to cause dose-dependent cytotoxicity for the human fibroblast-like fetal lung cell line MRC-5 [18], THP-1 monocytes [19], and HepG2 cells [20], which suggests that it is an “emerging mytotoxin”. In the latter study, Solhaug et al. reported that when added to HepG2 cells at sub-cytotoxic concentrations, AOD caused transient vacuolation, originating from lysosomes/late endosomes and associated with impaired endosomal/autophagic protein degradation due to acid lysosome dependence [20]. Thus, it appears that AOD exerts broad and complex effects on diverse cell types, but much more needs to be learned about its cellular effects, including potential alterations in metabolism and the activation of key signaling pathways.

In this study, we investigated the effects of AOD on cytotoxicity and gene expression in HepG2 cells. In addition, since we have recently found that AOD in contaminated food exists in the form of *N*-acyl metabolites [21], analogous to the *N*-acyl-sphingoid base backbones (“ceramides” and “1-deoxyceramides”) of mammalian sphingolipids, we also examined the effects of AOD on the sphingolipid composition of the cells as well as whether AOD undergoes acylation. The findings were that AOD or possibly its metabolites (*N*-acyl-AODs) affect the expression of multiple genes, including ones involved in cell stress and cellular sphingolipid metabolism.

## 2. Results

### 2.1. Effects of AOD, m18:0, and d18:0 on Cell Viability and the Cellular Content of Sphingoid Bases and AOD After Incubation with HepG2 Cells

**Figure 1:**
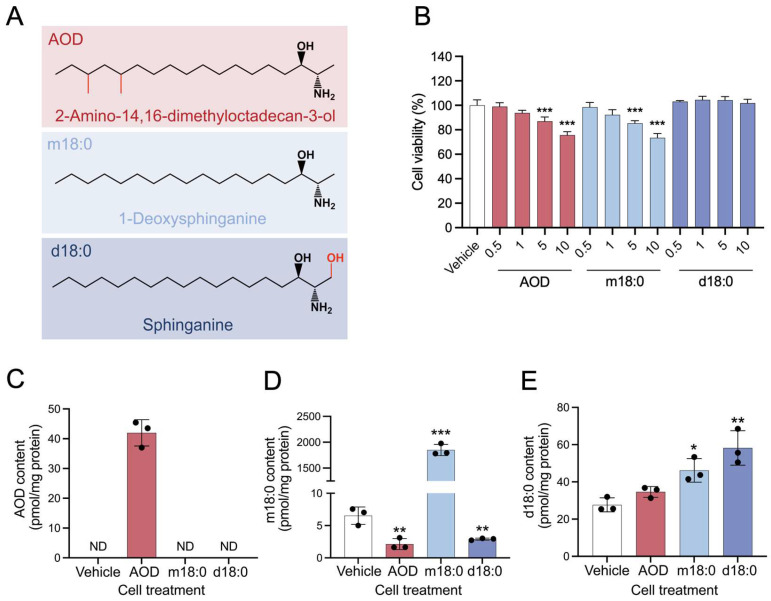
Structures of sphingoid bases and AOD, the effects of AOD, m18:0, and d18:0 on cell viability, and the cellular content of sphingoid bases and AOD after incubation with HepG2 cells. (**A**) Molecular structures of 2-amino-14,16-dimethyloctadecan-3-ol (AOD), 1-deoxysphinganine (m18:0), and sphinganine (d18:0) that are used in this study. (**B**) The viability of HepG2 cells was assessed by MTS assay after treatment with AOD, m18:0, or d18:0 (10 mM stock in ethanol diluted with complete medium to 0–10 µM) for 24 h. (**C**–**E**) Cellular levels of AOD, m18:0, and d18:0 were measured in HepG2 cells incubated with 5 µM of AOD, m18:0, or d18:0 for 24 h, followed by lipid extraction and sphingoid base measurement via LC-MS/MS. Data are mean ± SD and were analyzed by one-way ANOVA followed by Dunnett’s multiple comparisons test (**B**) and two-tailed unpaired *t*-tests (**C**–**E**). Significance levels are * *p* < 0.05, ** *p* < 0.01, and *** *p* < 0.001, with a sample size of *n* = 3.

Structurally, *Fusarium avenaceum* emerging mycotoxin AOD is highly homologous to atypical sphingoid base 1-deoxysphinganine (m18:0), and typical sphingoid base sphinganine (d18:0) (Figure 1A), both of which are endogenously synthesized in mammalian cells via the sphingolipid de novo synthesis [9]. Since free sphingoid bases [22,23] and AOD [19,20] are known to be cytotoxic, the first analysis was to determine if the levels used in our studies affected cell viability. The cell viability assay revealed that both AOD and m18:0 began to induce a significant decrease in cell viability at 5 μM when compared with cells treated with no sphingoid base or d18:0 (Figure 1B). A concentration of 5 μM was used for further investigations as it elicited significant physiological perturbations induced by AOD and m18:0 while maintaining cell viability above 85%, thereby mitigating substantial cell death from cytotoxicity.

The amounts of cellular AOD, m18:0, and d18:0 were determined after 24-h treatment with these compounds at 5 μM (or the ethanol vehicle) under the same conditions described above. The AOD amount in cells treated with 5 μM AOD was 42 ± 1.8 pmol/mg protein (Figure 1C), which was much lower (by 40-fold) than the amount of m18:0 in cells treated with 5 μM m18:0 (Figure 1D) and comparable to the amounts of d18:0 in cells treated with 5 μM d18:0 (Figure 1E). Interestingly, while having no significant effect on the level of endogenous d18:0 (*c.f.* AOD vs. vehicle in Figure 1E), as has been seen before [24], AOD substantially reduced the level of endogenous m18:0 (*c.f.* AOD vs. vehicle in Figure 1D). These results clearly demonstrate that, despite their structural similarities, the addition of these three compounds to cells results in distinctly different cellular sphingoid base profiles.

### 2.2. AOD Elicits Limited Transcriptomic Changes While Inducing Senescence-Associated Pathways

**Figure 2:**
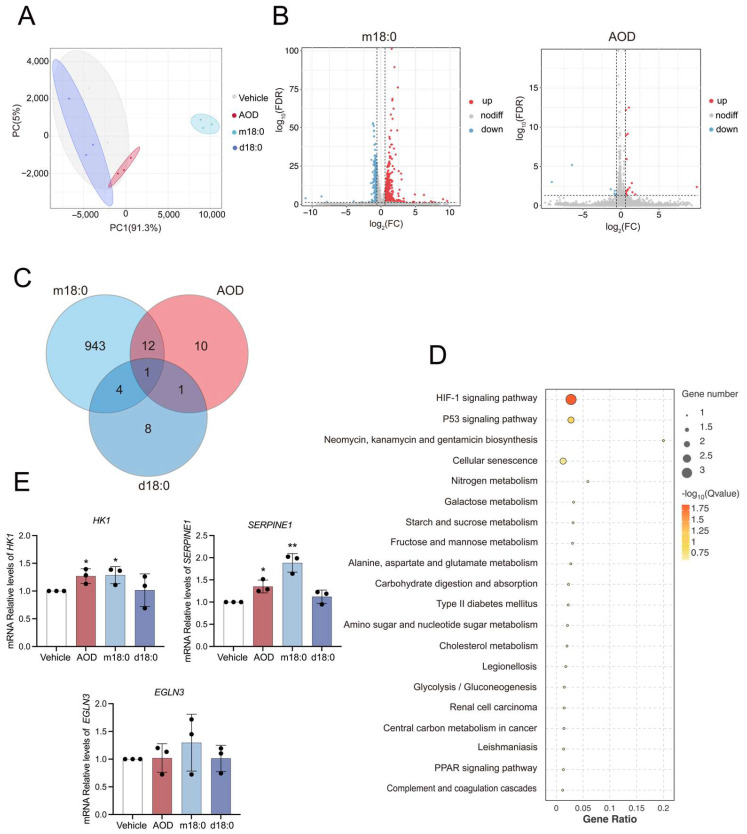
Distinct transcriptomic signatures of the effects of AOD and m18:0 versus d18:0 in HepG2 cells. (**A**) Principal component analysis (PCA) of RNA-seq samples from HepG2 cells treated with 5 μM AOD, m18:0, or d18:0 for 24 h (*n* = 3). (**B**) Volcano plot of RNA-seq data from HepG2 cells treated with m18:0 and AOD for 24 h. Genes with a fold change > 1.5 and an adjusted *p*-value < 0.05 are considered significantly differentially expressed. (**C**) Venn diagram of differentially expressed genes among AOD, m18:0, and d18:0 treatment groups. (**D**) KEGG enrichment analysis of common differentially expressed genes in AOD and m18:0 groups. (**E**) The mRNA expression levels of genes related to the HIF-1 signaling pathway, P53 signaling pathway, and cellular senescence signaling pathway. Data are mean ± SD and were analyzed by two-tailed unpaired *t*-tests. Significance levels are * *p* < 0.05 and ** *p* < 0.01, with a sample size of *n* = 3.

To further investigate the effects of AOD on cellular response, we deciphered the transcriptomic regulatory patterns by performing whole-genome RNA sequencing on cells treated with 5 μM AOD for 24 h. Meanwhile, to determine if the cellular responses from AOD stimulation are also caused by other exogenously added sphingoid bases, we examined m18:0 and d18:0 at the same concentrations. Principal component analysis (PCA) revealed that the first two principal components accounted for 96.3% of the variance (PC1 = 91.3%, PC2 = 5%). Notably, d18:0-treated cells clustered together with vehicle controls (Figure 2A), indicating AOD and m18:0 exhibited divergent gene regulatory modes versus d18:0.

Differential expression analysis (|fold change [FC]| ≥ 1.5; false discovery rate [FDR] < 0.05) identified 960 differentially expressed genes (DEGs) in m18:0-treated cells (648 upregulated; 312 downregulated), a number substantially higher than in the AOD (24 DEGs, with 17 upregulated and 7 downregulated) and d18:0 (14 DEGs, with 4 upregulated and 10 downregulated) groups (Figure 2B and Figure S1A). This quantitative disparity highlights m18:0-induced substantial transcriptional reprogramming compared to the limited transcriptional impact of AOD and d18:0. Notably, Venn diagram analysis revealed that AOD and m18:0 shared 13 core DEGs, far more than other pairwise comparisons (Figure 2C). KEGG pathway enrichment analysis showed that these shared genes were significantly enriched in pathways related to HIF-1 signaling (*HK1*, *SERPINE1*, and *EGLN3*), p53 networks (*SERPINE1* and *IGFBP3*), and cellular senescence (*SERPINE1* and *IGFBP3*). Pathways for neomycin, kanamycin, and gentamicin biosynthesis (*HK1*) and nitrogen metabolism (*CA9*) were not prioritized in this analysis, as both were enriched with only a single gene (Figure 2D).

The qRT-PCR validation of pathway-critical genes (*HK1*, *SERPINE1*, and *EGLN3*) demonstrated consistent upregulation trends in the AOD and m18:0 treatment groups (Figure 2E). Western blotting analysis revealed a significant accumulation of cleaved caspase-3 products (19 kDa) in AOD- and m18:0-treated cells, whereas d18:0 treatment exhibited only a marginal trend toward proteolytic activation (Figure S1B). Moreover, elevated levels of phosphorylated ERK in the AOD and m18:0 groups suggested potential activation of stress signaling pathways (e.g., MAPK/ERK cascade such as RAS-RAF-MEK-ERK, requiring further experimental validation), in contrast to the neutral effect of d18:0 (Figure S1B). These data indicate that AOD and m18:0 share a conserved ability to induce cellular stress responses. Remarkably, transcriptomic analysis revealed the activation of senescence-associated pathways, which was further validated by SA-β-gal staining showing a notable increase in SA-β-gal-positive cells in the AOD- and m18:0-treated groups (Figure S1C). Considering that human umbilical vein endothelial cells (HUVECs) typically require prolonged induction periods to develop robust senescence phenotypes [25], we implemented an extended treatment duration (72 h) with concomitantly reduced concentration (1 μM) to establish this cellular state. This finding offers preliminary evidence implying that AOD and m18:0 might affect cellular senescence pathways; nevertheless, further mechanistic investigation remains warranted.

### 2.3. Transcriptional Analysis of Genes Related to Sphingolipid Metabolism in AOD-Treated HepG2 Cells

**Figure 3:**
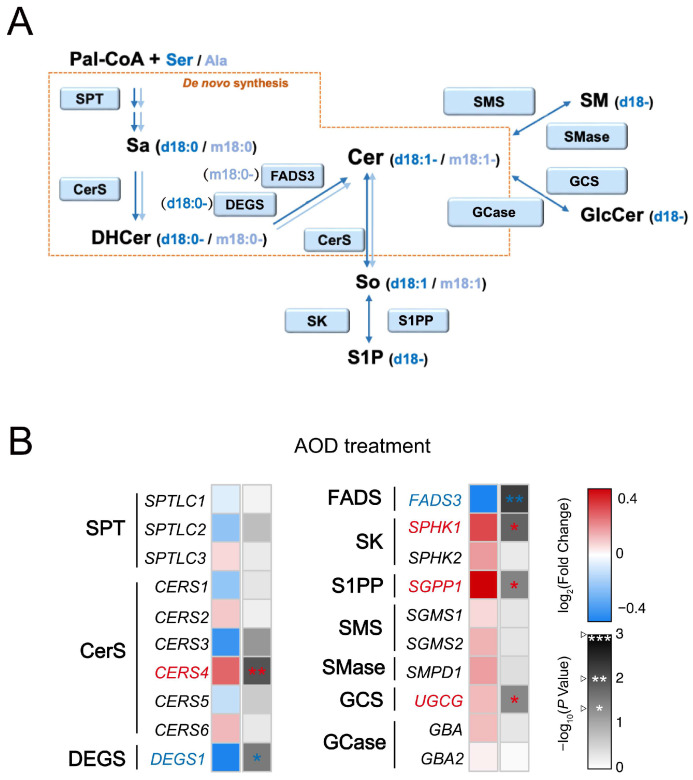
Effects of AOD mRNA expression levels of genes associated with the sphingolipid de novo synthesis pathway in HepG2 cells. (**A**) Sphingolipid synthesis pathway (only genes analyzed in (**B**) are shown). SPT, serine palmitoyltransferase(s); CerS, ceramide synthase(s); DEGS, dihydroceramide desaturase(s); FADS3, fatty acid desaturase 3; SK, sphingosine kinase(s); S1PP, S1P phosphatase(s); SMS, sphingomyelin synthase(s); SMase, sphingomyelinase(s); GCS, glucosylceramide synthase(s); GCase, glucocerebrosidase(s). (**B**) Heatmap depicting mRNA expression levels of genes associated with the sphingolipid synthesis pathway in HepG2 cells treated with AOD for 24 h versus vehicle controls. The red–white–blue color gradient represents log_2_-transformed fold changes (AOD/vehicle) with red indicating upregulation and blue denoting downregulation. Corresponding −log_10_ (*p*-values) are encoded in a white–black gradient (low to high). Data were analyzed by two-tailed unpaired *t*-tests. Significance levels are * *p* < 0.05, ** *p* < 0.01, and *** *p* < 0.001, with a sample size of *n* = 3.

Both m18:0 and d18:0 can be endogenously synthesized and undergo distinct further metabolism in the sphingolipid biosynthetic pathway (Figure 3A). Transcriptional analysis of genes related to sphingolipid metabolism showed that treatment with AOD selectively increased the mRNA expression of ceramide synthase 4 (*CERS4*) and sphingosine-1-phosphate phosphatase 1 (*SGPP1*), induced only a slight upregulation of UDP-glucosylceramide glucosyltransferase (*UGCG*), and produced a non-significant upward trend for sphingomyelin phosphodiesterase 1 (*SMPD1*), while simultaneously decreasing the expression of dihydroceramide desaturase 1 (*DEGS1*) and fatty acid desaturase 3 (*FADS3*) (Figure 3B). Intriguingly, a comparative analysis of gene expression profiles revealed a high degree of similarity between the effects of AOD and m18:0 treatments on these regulatory targets, but with a notable divergence from the effects of d18:0 treatment (Figure 3B and Figure S2).

### 2.4. Sphingolipid Profile Remodeling of HepG2 Cells in Response to AOD and Comparison with m18:0 or d18:0 Treatment

There are two reasons to suspect that AOD will alter the sphingolipid composition of cells: it has structural similarity to the *Fusarium* mycotoxin Fumonisin B1 (FB1) as well as sphingoid bases, which might mean that it acts as an inhibitor or substrate for enzyme(s) of this pathway, and the findings in the preceding section of this paper that AOD alters the expression of genes of the sphingolipid pathway might indicate that flux through the pathway has been altered at that level.

**Figure 4:**
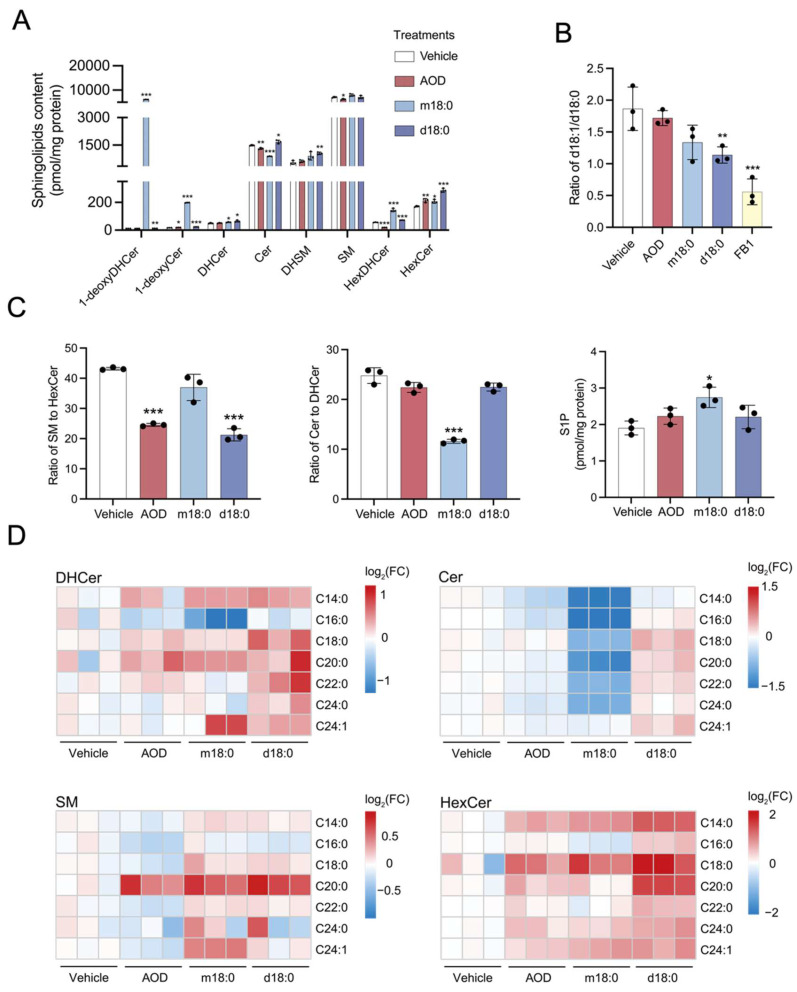
Sphingolipid profiles remodeling in response to AOD, m18:0, or d18:0 treatment. (**A**) The contents of various sphingolipids in HepG2 cells after 24-h treatment with 5 μM AOD, m18:0, or d18:0. (**B**) The ratio of the free sphingoid bases d18:1 to d18:0 after 24 h treatment with 5 μM AOD, m18:0, or d18:0. (**C**) The ratio of sphingomyelin (SM) to hexosylceramide (HexCer), the ratio of ceramide (Cer) to dihydroceramide (DHCer), and the content of sphingosine-1-phosphate in HepG2 cells after 24 h treatment with 5 μM AOD, m18:0, or d18:0. (**D**) Distribution of different acyl chain lengths of dihydroceramide, ceramide, sphingomyelin, and hexosylceramide in HepG2 cells after 24 h treatment with 5 μM AOD, m18:0, or d18:0. Data are mean ± SD and were analyzed by two-tailed unpaired *t*-tests (**A**–**C**). Significance levels are * *p* < 0.05, ** *p* < 0.01, and *** *p* < 0.001, with a sample size of *n* = 3.

The levels of major sphingolipid classes (e.g., ceramides [Cer], sphingomyelins [SM], etc.) in HepG2 cells treated with 5 μM AOD, or for comparison with 5 μM m18:0 or d18:0, were determined. While the total amounts of each sphingolipid class showed relatively minor changes in response to AOD addition at this concentration (Figure 4A), subtle differences can be seen by examining key metabolic nodes.

First, to preliminarily assess whether AOD exerts a similar effect on ceramide synthases as FB1 [26,27,28], we evaluated the ratio of free sphingoid bases d18:1 to d18:0 (Figure 4B) since fumonisins typically elevate d18:0 because addition of the double bond of d18:1 occurs after *N*-acylation (see Figure 3A). The results showed that, as expected, FB1 caused a large decrease in the d18:1/d18:0 ratio (Figure 4B), whereas AOD treatment, despite its structural resemblance to FB1, had no significant effect on this ratio. Exogenous m18:0 and d18:0 had small effects on the d18:1/d18:0 ratio, possibly because they are competing for endogenous substrates.

Next, one notices a change in the sphingomyelin/hexosylceramide (SM/HexCer) ratio (Figure 4C). This indicates that within the complex sphingolipid network, the metabolic flux has been redistributed, favoring the biosynthesis of glycosphingolipids over the synthesis of sphingomyelin. This might be attributable to its transcriptional activation of UDP-glucosylceramide glucosyltransferase (*UGCG*) (Figure 3B). Alternatively, this change may also involve the perturbation of the hydrolysis process of complex sphingolipids. Such a metabolic alteration implies that the flux of sphingoid bases tends to be directed more towards the synthesis of glucosylceramide.

Additionally, AOD treatment also caused a small but statistically significant decrease in the total cellular ceramide levels (Figure 4A). Ceramide synthase 4 (CerS4) exhibits substrate preference toward fatty acyl-CoAs with C18 and C20 acyl chains [29,30]. Analysis of the *N*-acyl chain-length distribution revealed that in the AOD-treated group, there was a selective enrichment of C18:0 and C20:0 dihydroceramide (DHCer) species (Figure 4D), a pattern that may be consistent with the previously observed upregulation of the expression of ceramide synthase 4 (*CERS4*) mRNA (Figure 3B).

When treated with the same concentration, exogenous d18:0 also significantly reduced the sphingomyelin/hexosylceramide (SM/HexCer) ratio in cells, which was distinct from that of the m18:0-treated group. The exogenous m18:0 treatment specifically decreased the ceramide/dihydroceramide (Cer/DHCer) ratio, which is mechanistically in line with its previously demonstrated ability to suppress the expression of dihydroceramide desaturase 1 (*DEGS1*) mRNA (Figure S2). In contrast, neither the d18:0-treated group nor the AOD-treated group showed significant changes in the ceramide/dihydroceramide (Cer/DHCer) ratio. Simultaneously, the m18:0 treatment uniquely increased the level of sphingosine-1-phosphate (S1P), which was correlated with the upregulated expression of sphingosine kinase 1 (*SPHK1*) mRNA (Figure S2), while the S1P concentration remained stable in other treatment groups (Figure 4C). Notably, the exogenous m18:0 also significantly reduced the total cellular ceramide levels and exhibited a selective enrichment of C18:0 and C20:0 dihydroceramide (DHCer) species, which was the same as the phenomenon observed after AOD treatment (Figure 4D). Although AOD treatment upregulated *SPHK1* mRNA, it concurrently induced a more significant upregulation of sphingosine-1-phosphate phosphatase 1 (*SGPP1*) (Figure 3B), potentially explaining the stable S1P levels observed in the AOD-treated group. This indicates that AOD and m18:0 demonstrate coordinated regulation in terms of ceramide abundance and acyl chain specificity. Moreover, all experimental groups showed a substantial accumulation of dihydro-complex sphingolipids, dihydrosphingomyelin (DHSM), and dihydrohexosylceramide (HexDHCer) (Figure S3).

### 2.5. AOD Is Metabolized into 1-Deoxydihydroceramide-like Molecules, and This Is Inhibited by Fumonisin B1

**Figure 5:**
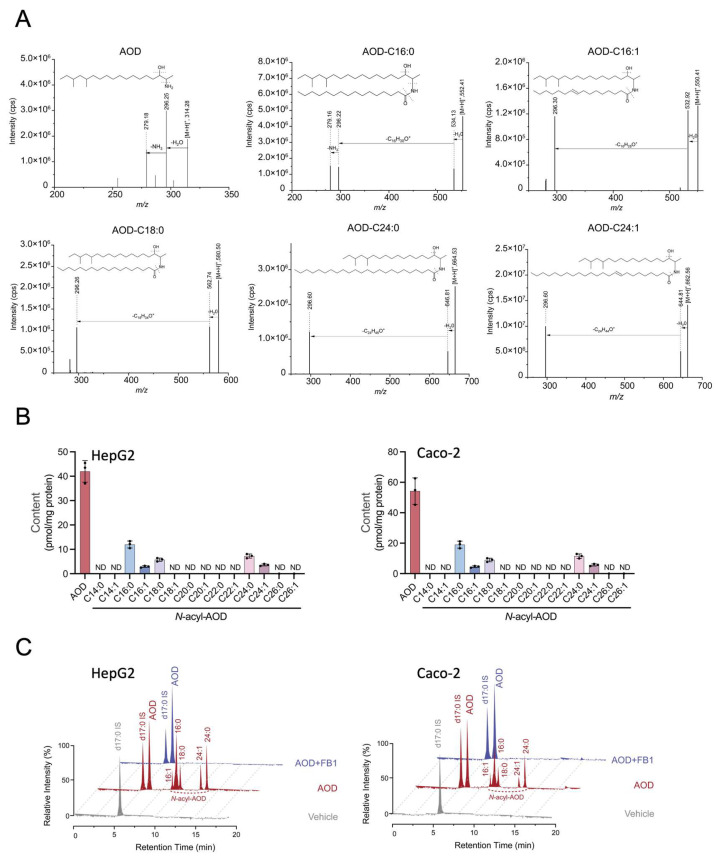
AOD is metabolized to 1-deoxyceramide-like molecules unless inhibited by FB1. (**A**) Mass spectrometric analysis of AOD and *N*-acyl-AODs in positive electrospray ionization mode. Shown here are the fragments from collisionally induced dissociation of the precursor ions for AOD and AOD with the shown *N*-acyl chains that were extracted from HepG2 cells incubated with 5 μM AOD for 24 h. (**B**) The relative amounts of AOD and *N*-acyl-AOD in HepG2 or Caco-2 cells treated with AOD for 24 h. (**C**) The selected ion chromatograms of AOD and *N*-acyl-AOD were analyzed by LC-MS/MS after the HepG2 or Caco-2 cells were treated with 5 μM AOD and 10 μM FB1 for 24 h. Data are mean ± SD, with a sample size of *n* = 3 (B). IS denotes the internal standard.

Based on the structural homology between AOD and sphingoid bases (d18:0 and m18:0; Figure 1A), we hypothesized that this sphingoid base analog might enter sphingolipid metabolic pathways. To explore the metabolism of AOD in mammalian cells, targeted lipidomic profiling was performed to trace its *N*-acylated derivatives. By applying our previously established LC-MS/MS analysis [21], we detected five AOD derivatives through their characteristic fragmentation patterns: C16:0, C16:1, C18:0, C24:0, and C24:1 *N*-acyl-AOD (Figure 5A). Metabolomic analysis of HepG2 cells after 24-h exposure to AOD revealed the relative distribution of AOD among these *N*-acyl-AODs, with parallel analysis in Caco-2 cells yielding similar abundance profiles (Figure 5B), suggesting potential conservation of this metabolic process in the tested cell lines.

To explore if ceramide synthases (CerS) might be involved in AOD *N*-acylation, we pre-treated cells with 10 μM FB1 to inhibit CerS. With FB1 pre-treatment, the *N*-acyl-AOD could no longer be detected in either HepG2 or Caco-2 cells (Figure 5C). This suggests that CerS may be responsible for the *N*-acylation of AOD.

Although the amounts of AOD and its metabolites in the cells are only estimates due to the lack of definitive internal standards for quantification, it appears that only a fraction of the AOD in the cells appears as the *N*-acyl species (Figure 5C). Furthermore, the sum of AOD and these metabolites (from the data in Figure 1C and Figure 5C) represents about 1% of the AOD provided to cells in the culture medium, which is much lower than the percentage of m18:0 that appears to be taken up (~half of the administered amount, calculated from Figure 1D and Figure 4A).

## 3. Discussion

In this study, we found that AOD reshapes some aspects of the transcriptome and sphingolipidome of mammalian cells, adding to the effects that AOD has previously been reported to have on THP-1 monocytes [19] and HepG2 cells [20]. Our additional discovery that AOD undergoes *N*-acylation by mammalian cells extends our previous identification of “ceramide-mimic” *N*-acyl-AOD molecules in *F. avenaceum*-fermented rice cultures [21]. Although these findings necessitate a more detailed mechanistic follow-up, the convergent evidence from gene expression profiles and metabolic remodeling confirms AOD’s significance as an emerging mycotoxin, warranting further investigation. Therefore, future studies of both AOD and its *N*-acyl metabolites will be essential for understanding its cellular effects as well as its occurrence in food.

It is also significant that AOD begins to show cytotoxicity and multiple other cellular effects at a concentration (5 μM) where it has undergone a much lower cellular uptake (~1%) than what occurs with exogenous m18:0 (~50%) when added to the medium at the same concentration. This differential uptake may partially account for the disparity in the number of differentially expressed genes affected by AOD and m18:0. This observation also suggests that whatever the targets of AOD are, its effects on stress-related gene transcription appear to be potent, which is potentially attributable to high-affinity interactions with molecular targets. The lower uptake of AOD might be due to differences in the solubility of AOD versus m18:0 in the culture medium due to the two additional methyl groups on the AOD side chain, or possibly to a more interesting mechanism such as differences in uptake or efflux transporter(s) for sphingoid bases [31]. Further research is needed to elucidate this. While we did not investigate this as part of our study, it is likely that the absence of substantial accumulation of d18:0 was not due to poor uptake, but rather, rapid metabolic clearance (irreversible degradation or incorporation into more complex sphingolipids) that is not feasible for 1-deoxy sphingoid bases such as AOD and m18:0.

Transcriptomic profiling further corroborated the mechanistic divergence among analogs. Pathway enrichment analysis specifically linked AOD and m18:0, but not d18:0, to HIF-1, p53, and cellular senescence signatures—all hallmarks of stress-responsive adaptation. These associations suggest that AOD and m18:0 may trigger specific cellular stress responses, which could have significant implications for cell survival and function. Solhaug et al. demonstrated [20] that AOD induces non-lethal vacuolization in HepG2 cells through impaired lysosomal degradation and disrupted autophagy, likely as a stress adaptation response. In their earlier investigations [19], AOD also exhibited dose-dependent effects in THP-1 cells, altering metabolic activity, cell death dynamics, and cell cycle progression. These findings substantiate AOD’s capacity to elicit broad stress responses that compromise cellular homeostasis.

Sphingolipid flux divergence was particularly evident in AOD-treated cells, where metabolic routing toward glycosphingolipid biosynthesis correlated with *UGCG* upregulation. Meanwhile, the observed decline in SM and Cer levels merits attention. As essential constituents of the plasma membrane, SM and ceramide not only maintain structural integrity but also orchestrate critical cellular functions, including signal transduction through lipid raft formation, ion channel gating modulation, and protein sorting machinery [32]. These alterations may underpin potential mechanisms by which AOD influences cellular homeostasis. It cannot yet be concluded that changes in sphingolipid-related genes account for the differences in sphingolipid levels in AOD-treated cells. Future studies should also consider other factors, such as the production and turnover of metabolites in alternative pathways, as well as their intracellular trafficking and efflux from cells. While m18:0 elevated S1P levels via sphingosine kinase activation—a phenomenon absent in AOD-treated cells—both analogs shared conserved ceramide-lowering effects. This functional convergence, coupled with structural homology to the cytotoxic m18:0 derivative 1-deoxydihydroceramide [11,33,34,35], suggests overlapping mechanisms in cellular response modulation. Previous studies indicate that endogenous accumulation of m18:0, driven by serine deprivation, triggers proteolysis of sphingosine kinase, thereby reducing the level of sphingosine-1-phosphate (S1P) [36]. In contrast, our experiments demonstrate that exogenous supplementation of m18:0 elicits opposing effects. We speculate that this discrepancy arises from the mechanistic differences between endogenous synthesis and exogenous administration: excess extracellular m18:0 may compete with canonical sphingoid bases for the binding sites of ceramide synthase (CerS), thereby reducing intracellular ceramide production. Consequently, the accumulated free sphingoid bases may be diverted toward S1P synthesis via the sphingosine kinase. We observed elevated levels of C20:0 dihydroceramide (DHCer) and sphingomyelin (SM) across the AOD, m18:0, and d18:0 treatment groups. Previous studies have demonstrated that exogenous lipids can effectively displace phospholipids in the outer leaflet of the cytoplasmic membrane [37], potentially expanding the intracellular pool of bioavailable free fatty acids. We hypothesize that AOD, m18:0, and d18:0 may enhance intracellular levels of C20 acyl-CoA substrates, thereby facilitating the synthesis of C20:0 SM and DHCer in all treatment groups, which requires further validation. It is interesting that the comparison of these three sphingoid bases found some similarities and some differences, but a more in-depth investigation is needed to determine if they are due to differences in cellular uptake, metabolism, and/or structure-specific effects on membrane biophysics or signaling targets.

In summary, AOD is taken up by mammalian cells and subsequently undergoes *N*-acylation (likely mediated by CerS, which requires further verification through in vitro enzyme activity assays) to form ceramide analogs, concomitant with a reduction in endogenous ceramide levels. Critically, it remains unclear whether the cellular effects induced by AOD treatment arise from its direct action or its *N*-acylated metabolites, warranting further investigation to elucidate their distinct contributions. At the transcriptomic level, AOD induces activation of stress-responsive pathways, including the hypoxia-inducible factor-1α (HIF-1α) signaling pathway, the p53 signaling pathway, and cellular senescence. These coordinated responses are consistently observed in m18:0-treated cells but remain undetectable in d18:0-exposed counterparts. The causal interplay between AOD-induced sphingolipidomic remodeling and transcriptomic reprogramming remains undefined. To clarify this connection, future studies could employ targeted metabolic interventions: for instance, using ceramide synthase inhibitors to block endogenous ceramide synthesis, or performing rescue experiments with exogenous sphingolipids to assess whether restoring specific lipid species mitigates transcriptional changes. This knowledge gap necessitates further studies to elucidate whether AOD’s transcriptional effects are driven by sphingolipid perturbation and whether metabolic interventions could mitigate its cellular impact. Clarifying these mechanisms could reveal key drivers of AOD toxicity and inform strategies to assess whether it warrants classification as a mycotoxin—requiring further evidence such as in vivo confirmation of organ-specific toxicity, stability in food matrices during processing, and establishment of dose–response relationships for human health risk assessment.

## 4. Materials and Methods

### 4.1. Reagents

1-deoxysphinganine (m18:0) (Avanti Polar Lipids, Alabaster, AL, USA, #860493) and sphinganine (d18:0) (Avanti Polar Lipids, Alabaster, AL, USA, #860498) were obtained in powder form. 2-amino-14,16-dimethyloctadecan-3-ol (AOD) was prepared and purified according to a method previously established in our laboratory [21]. Briefly, *Fusarium avenaceum* was first cultured on a synthetic agar low in nutrients (Synthetischer Nährstoffarmer Agar, SNA, Solarbio, Beijing, China, #LA8940), before being transferred to a rice medium. The lyophilized culture was extracted with methanol, partitioned between water and diethyl ether, and the ether phase was processed. The residue was separated by thin-layer chromatography using a chloroform/methanol/water (60:40:1, *v*/*v*/*v*) solvent system, with the target AOD fraction identified via iodine staining (referenced to m18:0) and collected. These chemicals were dissolved in ethanol at a concentration of 10 mM to prepare stock solutions, which were stored at −20 °C for no longer than 7 days. To ensure analytical accuracy, working solutions were prepared fresh daily using aliquoted solutions. The LIPID MAPS™ internal standard cocktail (Sphingolipid Mix II, #LM-6005) was from Avanti Polar Lipids, Alabaster, AL, USA, and was certified to be over 95% pure and within 10% of the specified amount (25 μM). The HPLC-grade methanol (Sigma, St. Louis, MO, USA, #34860), 2-propanol (Merck, Darmstadt, Germany, #1.01040), formic acid (Macklin, Shanghai, China, #809712), chloroform (Sigma, USA, #39918), dichloromethane (Sigma, USA, #34856), and acetonitrile (Sigma, St. Louis, MO, USA, #34851) used for lipid extraction and analysis by liquid chromatography coupled to electrospray ionization tandem mass spectrometry (LC−MS/MS) analysis were obtained from Sigma (St. Louis, MO, USA), Macklin (Shanghai, China), and Merck (Darmstadt, Germany). The ammonium formate (Sigma, St. Louis, MO, USA, #70221) and ammonium acetate (Sigma, St. Louis, MO, USA, #73594) were from Sigma (St. Louis, MO, USA).

### 4.2. Cell Culture Preparation and Treatment

The HepG2 and Caco-2 cells used for the experimental analyses, obtained from the Cell Bank of the Chinese Academy of Sciences (Kunming, China, #KCB200507YJ, #KCB200710YJ), were cultured in Dulbecco’s Modified Eagle Medium (DMEM, Corning, New York, NY, USA, #10-013) supplemented with 10% heat-inactivated fetal bovine serum (FBS, Clarke Bioscience, Houston, TX, USA, #FB25015) and 100 U/mL penicillin–streptomycin (PS, Solarbio, Beijing, China, #P1400). The cultures were maintained at 37 °C in a humidified incubator with a 5% CO_2_ atmosphere. Cells were passaged using 0.25% trypsin (Solarbio, Beijing, China, #T8150) to detach adherent cells from the culture dish surface during subculturing.

HepG2 cells were seeded in six-well plates at a density of 1.0 × 10^6^ cells per well and allowed to adhere for 12 h. Except where otherwise specified, the cells were subsequently treated with AOD, m18:0, or d18:0 at a final concentration of 5 µM for 24 h. The compounds were added to a complete medium (DMEM supplemented with 10% FBS and 1% penicillin–streptomycin) from the stock solutions in ethanol immediately prior to treatment. The final concentration of ethanol was 1 µL per 2 mL of the medium (0.5%), and control wells were treated with an equivalent volume of ethanol alone. Fumonisin B1 (FB1, MCE, Monmouth Junction, NJ, USA, #116355-83-0) was applied at a final concentration of 10 µM, prepared from a 10 mM stock solution in DMSO (Solarbio, Beijing, China, #D8371). Cells were pre-treated with FB1 for 2 h prior to the addition of the indicated compounds, followed by a 24 h co-incubation with both FB1 and the compounds. Unless otherwise specified, all treatments were conducted under the same culture conditions for 24 h.

### 4.3. Cell Viability Assay

HepG2 cells were seeded in 96-well plates containing 100 µL of the medium at a density of 1.0 × 10^5^ cells per well and allowed to adhere for 12 h. Subsequently, cells were treated with increasing concentrations (0–10 μM) of AOD, m18:0, or d18:0 for 24 h. The compounds were initially dissolved in ethanol to prepare stock solutions, followed by dilution in a complete medium immediately prior to treatment (the final volume of ethanol was ≤1 µL/mL medium). Following treatment, the MTS reagent (CellTiter 96^®^ AQueous One Solution, Promega, Madison, WI, USA, #G3580) was diluted in a serum-free medium according to the manufacturer’s protocol. Then, 100 μL of this mixture was added to each well, and plates were incubated at 37 °C for 1 h. Absorbance was measured at 490 nm using Flexstation3 (Molecular Devices, San Jose, CA, USA). Cell viability was normalized to vehicle-treated controls and calculated as follows: viability (%) = [(OD treatment − OD blank)/(OD vehicle − OD blank)] × 100.

### 4.4. Lipid Extraction

Cells cultured in 6-well plates were harvested at 80–90% confluence. After two washes with phosphate-buffered saline (PBS) to remove residual culture medium, the cells were detached using a cell scraper. The harvested cells were then resuspended in 150 μL of double-distilled water to prepare a homogeneous cell suspension. Lipids were extracted using a previously described method with slight modifications [21].

For sphingoid bases and sphingosine-1-phosphate extraction, sample suspensions were transferred into 13 × 100 mm screw-capped glass test tubes with Teflon caps. Sequential additions of 1 mL methanol, 0.5 mL dichloromethane, and 1 μL internal standard (Avanti Polar Lipids, Alabaster, AL, USA, #LM 6005) were performed. The mixtures were vortex-mixed (30 s), sonicated (5 min), and incubated overnight at 48 °C. After cooling to room temperature, 150 μL of 1 M methanolic potassium hydroxide was added, followed by brief vortex agitation and a 2 h incubation at 37 °C. Neutralization was achieved by adding 8 μL of glacial acetic acid, and the neutral pH was confirmed by test strips. Following centrifugation (1000 rpm, 10 min), the supernatant was carefully collected using glass Pasteur pipettes. The residual phase was re-extracted twice with 1 mL of methanol:dichloromethane (2:1, *v*/*v*). Combined phases were vacuum-dried and reconstituted in 250 μL of methanol:dichloromethane (2:1, *v*/*v*). After centrifugation (12,000 rpm, 10 min), 2 μL of the supernatant was analyzed by LC-MS/MS.

For ceramides and complex sphingolipids, extraction reagents were modified to 1 mL of methanol and 0.5 mL of chloroform, with subsequent steps mirroring the initial protocol. Following neutralization with 6 μL of glacial acetic acid and the addition of 2 mL of double-distilled water, the test tubes were centrifuged (1000 rpm, 10 min) to facilitate collection of the lower chloroform phase. The residual phase underwent triple re-extraction with pure chloroform (1 mL, 3 times). Combined organic phases were dried, and the residues were reconstituted in 250 μL of methanol:chloroform (2:1, *v*/*v*), centrifuged (12,000 rpm, 10 min), and analyzed by LC-MS/MS using 2 μL of the supernatant.

### 4.5. LC-MS/MS Analysis

The analysis of sphingolipids, including AOD and its *N*-acyl metabolites, followed our previous work with slight modifications [21,38]. All sphingolipids were analyzed using an ultra-high-performance liquid chromatography (UPLC) system (Shimadzu, Kyoto, Japan) coupled to a Triple Quad™ 5500+ QTRAP™ mass spectrometer (AB Sciex, Framingham, MA, USA), with a total of 76 molecules (excluding internal standards) being targeted. The UPLC system consisted of a binary pump (LC-30AD), degasser (DGU-20A5), temperature-controlled autosampler (SIL-30AC), column oven (CTO-20AC), and system controller (CBM-20A). The mass spectrometer was operated in positive electrospray ionization (ESI+) mode with scheduled multiple reaction monitoring (MRM) transitions, as detailed in Supplementary Table S1. Ion source parameters were set as follows: ESI voltage at 4.5 kV, source temperature at 40 °C, ion source gas 1 (nebulizer) at 60 psi, ion source gas 2 (heater) at 40 psi, and curtain gas flow at 40 psi. Dwell time and inter-channel delay were configured to 50 ms and 5 ms, respectively. Instrument control and data acquisition were managed using Analyst 1.7.3 software (Applied Biosystems, Foster City, CA, USA). The sphingolipid quantities were normalized to protein concentration determined by the Pierce BCA Protein Assay Kit (Thermo Scientific, Waltham, MA, USA, #23225). The approximate quantities of AOD and its *N*-acyl derivatives were estimated from standard curves prepared using the m18:0 sphingoid base standard (Avanti Polar Lipids, Alabaster, AL, USA, #860493) and d18:1-C12:0 ceramide internal standard from the Cer/Sph Mixture II (Avanti Polar Lipids, Alabaster, AL, USA, #LM 6005), respectively.

Chromatographic separation of sphingoid bases and sphingosine-1-phosphate (S1P) was performed on a C18 column (2.6 μm, 100 × 2.1 mm, 100 Å, Phenomenex, Torrance, CA, USA) with mobile phase A (methanol:water:formic acid = 58:41:1, 5 mM ammonium formate) and mobile phase B (methanol:formic acid = 99:1, 5 mM ammonium formate). The column was equilibrated with 50% mobile phase B for 2 min prior to injection. The gradient program was performed as follows: 0–1 min at 25% B, 1–3 min linear increase to 100% B, 3–5 min holding at 100% B, 5–5.8 min returning to 25% B, and 5.8–6 min re-equilibration at 25% B. The flow rate was maintained at 0.5 mL/min with a 2 μL injection volume.

For ceramides and complex sphingolipids, the same C18 column was used with mobile phase A (methanol:water:acetonitrile = 1:1:1, 7 mM ammonium acetate) and mobile phase B (isopropanol, 7 mM ammonium acetate). After equilibration with 50% B for 2 min, the gradient elution was carried out as follows: 0–1 min at 50% B, a linear gradient from 50% to 98% B over 1–8 min, holding at 98% B from 8 to 11 min, returning to 50% B from 11 to 11.1 min, and re-equilibration at 50% B from 11.1 to 12 min. The flow rate and injection volume were set to 0.3 mL/min and 2 μL, respectively.

For the *N*-acylated derivatives of AOD, the same C18 column and mobile phase system were employed as those used for ceramides. The column was equilibrated with 50% mobile phase B for 2 min prior to injection. The gradient elution program consisted of 0–2 min at 25% B; 2–6 min linear gradient from 25% to 100% B; 6–18 min isocratic elution at 100% B; 18–19 min linear return to 25% B; and 19–20 min re-equilibration at 25% B. The mobile phase flow rate was maintained at 0.5 mL/min with a 2 μL injection volume.

Quantification of sphingolipid analytes was performed by integrating peak areas for analytes and internal standards using SCIX OS (Version 3.0.0.3339, Applied Biosystems, Foster City, CA, USA), followed by calculation using the following formula: picomoles of analyte = correction factor × (peak area of each analyte/peak area of internal standard) × picomoles added internal standards. Correction factors for C16:0, C18:1, C20:0, C24:0, and C24:1 species were determined directly from standards versus their designated internal standards (C12 Cer, C12 SM, and C12 GluCer); factors for C18:0, C20:1, C22, C26 species were derived from C12, C16:0, C18:1, C20:0, C24:0, and C24:1 standards based on chain-length regularities [39]; sphingoid bases and sphingosine-1-phosphate factors were obtained directly using standards versus d17:0, d17:1, or S1P-d17:1 internal standards; and AOD with its *N*-acyl metabolites used factors for m18:0 and 1-deoxydihydroceramide.

### 4.6. Cellular RNA Sequencing

Total RNA was isolated from HepG2 cells cultured in 6-well plates and harvested at 80–90% confluence and subjected to RNA library sequencing on the Illumina NovaSeq X Plus platform by Gene Denovo Biotechnology Co., Ltd. (Guangzhou, China). Raw sequencing data underwent rigorous quality control, including adapter trimming, filtration of low-quality reads (Q-score ≤ 20), and base-level quality assessment, to ensure the accuracy of subsequent analyses. Transcript expression levels were quantified as transcripts per million (TPM) values, which were used for further bioinformatic analysis. Bioinformatic analyses were performed using Omicsmart, a real-time interactive online platform for data analysis (http://www.omicsmart.com, accessed on 14 August 2025).

### 4.7. Quantitative Real-Time PCR

Total RNA was extracted from cells cultured in 6-well plates at 80–90% confluence using RNAiso Plus (TaKaRa, Kusatsu, Japan, #9108) and quantified with a NanoDrop One spectrophotometer (Thermo Fisher, Waltham, MA, USA). The cDNA synthesis was conducted with 1 μg of RNA using the PrimeScript™ RT reagent kit (TaKaRa, Kusatsu, Japan, #RR047A). Quantitative PCR (qPCR) was performed with 2× M5 HiPer Realtime PCR Supermix (Mei5 Biotechnology, Beijing, China, #MF013-01). Relative gene expression was determined by the 2^−ΔΔCt^ method, with primer sequences provided in Supplementary Table S2.

### 4.8. Western Blotting

HepG2 cells cultured in 6-well plates and harvested at 80–90% confluence were lysed in a RIPA buffer (Solarbio, Beijing, China, #R0010) supplemented with a protease inhibitor cocktail (Roche Applied Science, Basel, Switzerland, #4693159001) and a phosphatase inhibitor cocktail (Roche Applied Science, Switzerland, #04906837001) on ice for 15 min. Lysates were centrifuged at 13,000× *g* for 10 min at 4 °C, and the protein concentration was determined using the Bicinchoninic Acid Assay (Thermo Fisher, USA, #A55864). Protein samples were normalized to a concentration of 2 mg/mL. Equal amounts of protein (30 μg) were separated by 12.5% sodium dodecyl sulfate–polyacrylamide gel electrophoresis (SDS-PAGE) at 120 V and subsequently transferred to polyvinylidene difluoride (PVDF) membranes under a constant current of 200 mA.

The membranes were blocked with 5% skimmed milk for 1 h at room temperature and then incubated overnight at 4 °C with the following primary antibodies: GAPDH (Proteintech, Wuhan, China, #60004-1-Ig, 1:5000), pERK (Cell Signaling Technology, Danvers, MA, USA, #4695, 1:1000), ERK (Santa Cruz Biotechnology, Dallas, TX, USA, #sc-135900, 1:200), and Caspase-3 (Santa Cruz Biotechnology, Dallas, TX, USA, #sc-56053, 1:200). After washing three times with phosphate-buffered saline tween-20 (PBST), the membranes were incubated with horseradish peroxidase (HRP)-conjugated secondary antibodies at room temperature for 1 h. Protein signals were visualized using an enhanced chemiluminescence (ECL) kit (Tanon, Shanghai, China, #180-5001) and captured using an image analysis system. Gray values were quantified using ImageJ software(Version 1.52p, National Institutes of Health, Bethesda, MD, USA).

### 4.9. SA-β-Gal Staining

Human umbilical vein endothelial cells (HUVECs) were stained for senescence-associated β-galactosidase (SA-β-gal) using a commercial kit (Beyotime Biotechnology, Shanghai, China, #C0602). HUVECs were seeded in 6-well plates at a density of 1.0 × 10^6^ cells per well and treated with 1 μM d18:0, m18:0, or AOD for 3 days. After trypsinization (Solarbio, Beijing, China, #T8150), cells were re-seeded into 12-well plates at 30–40% confluency determined with a digital microscope. Following 6 h of attachment, the cells were washed three times with phosphate-buffered saline (PBS), fixed with fixation buffer at room temperature for 15 min, washed again three times with PBS, and incubated with the SA-β-gal staining buffer at 37 °C for 12 h. Senescence-positive HUVECs, identified by blue staining, were quantified under a microscope (ZEISS, Oberkochen, Germany).

### 4.10. Statistical Analysis

All experiments were performed with a sample size of *n* = 3 biological replicates, and data are presented as mean ± standard deviation (SD) unless otherwise specified. Statistical analyses were performed using Student’s *t*-tests or one-way ANOVA, and a *p*-value < 0.05 was considered statistically significant. All figures were generated using GraphPad Prism 10 (GraphPad Software, La Jolla, CA, USA) and R with ggplot2.

## Data Availability

The raw RNA sequencing (RNA-seq) data and bulk expression read counts generated in this study were deposited in the Genome Sequence Archive [40] in the National Genomics Data Center [41], China National Center for Bioinformation/Beijing Institute of Genomics, and Chinese Academy of Sciences (GSA-Human: HRA010920), which are publicly accessible at https://ngdc.cncb.ac.cn/gsa-human (accessed on 14 August 2025). Differential expression data are provided in the Supplementary Materials. Additional data are available upon request from the authors. Source data are included within this manuscript.

## Supplementary Materials

The following supporting information can be downloaded at https://www.mdpi.com/article/10.3390/toxins17080413/s1, Figure S1: Volcano plot of differentially expressed genes (d18:0-treated vs. vehicle control) and effects of AOD and m18:0 on cellular senescence and stress response signaling; Figure S2: The regulation of sphingolipid metabolism-related genes by d18:0 and m18:0; Figure S3: Targeted analysis of sphingolipids with differential *N*-acyl chain lengths by LC-MS/MS; Table S1: Ion transitions and parameters for LC-MS/MS analysis; Table S2: qPCR primer sequences used in this study.

## Abbreviations

The following abbreviations are used in this manuscript.

AOD	2-amino-14,16-dimethyloctadecan-3-ol
m18:0	1-deoxysphinganine
d18:0	sphinganine
d18:1	sphingosine
SPT	serine palmitoyltransferase(s)
CerS	ceramide synthase(s)
DEGS	dihydroceramide desaturase(s)
dhCerS	dihydroceramide synthase(s)
Cdase	ceramidase(s)
GCase	glucocerebrosidase(s)
GCS	glucosylceramide synthase(s)
SMS	sphingomyelin synthases(s)
SMase	sphingomyelinase(s)
SK	sphingosine kinase(s)
S1PP	S1P phosphatase(s)
*HK1*	hexokinase 1
*EGLN3*	egl-9 family hypoxia-inducible factor 3
*SERPINE1*	serpin family E member 1
*CERS4*	ceramide synthase 4
*DEGS1*	dihydroceramide desaturase 1
*FASD3*	fatty acid desaturase 3
*SPHK1*	sphingosine kinase 1
*SGPP1*	sphingosine-1-phosphate phosphatase 1
*UGCG*	UDP-glucosylceramide glucosyltransferase
S1P	sphingosine-1-phosphate
Cer	ceramide
SM	sphingomyelin
HexCer	hexosylceramide
DHCer	dihydroceramide
DHSM	dihydrosphingomyelin
HexDHCer	dihydrohexosylceramide
FB1	fumonisin B1
PCA	principal component analysis
DEGs	differentially expressed genes
SNA	Synthetischer Nährstoffarmer Agar
LC–MS/MS	liquid chromatography tandem mass spectrometry
UPLC	ultra-high-performance liquid chromatography
sMRM	scheduled multiple reaction monitoring
TPM	transcripts per million
RNA-seq	RNA sequencing
PBS	phosphate-buffered saline
SA-β-gal	senescence-associated β-galactosidase
