# Supplementary material for: The Emerging Mycotoxin 2-Amino-14, 16-Dimethyloctadecan-3-ol (AOD) Alters Transcriptional Regulation and Sphingolipid Metabolism and Undergoes N-Acylation by HepG2 Cells
Source: Toxins (Basel). 2025 Aug 15;17(8):413. doi: 10.3390/toxins17080413 (PMC12390525; doi:10.3390/toxins17080413)
Supplement: Supplementary file 1 [file toxins-17-00413-s001.zip › toxins-3777410-supplementary.pdf]

Supplementary Materials: The Emerging Mycotoxin 2-Amino-14,16-dimethyloctadecan-3-ol (AOD) Alters Transcriptional Regulation and Sphingolipid Metabolism and Undergoes N-Acylation by HepG2 Cells

Shenlong Mo, Zhenying Hu, Huaiyi Zhu, Boming Yu, Xiaoyan Chen, Yu Chen, Alfred H. Merrill, Jr. and Jingjing Duan

Supplementary Table S1. Ion transitions and parameters for LC-MS/MS analysis

Metabolite	Parent ion (*m/z*)	Fragment ion (*m/z*)	Declustering Potential (V)	Collision energy (V)
d17:1	286.3	268.3	110	15
d17:0	288.3	270.3	100	15
S1P-d17:1	366.3	250.3	100	35
S1P-d17:0	368.3	270.3	100	20
m18:1	284.3	266.3	115	15
m18:0	286.3	268.3	70	25
d18:1	300.3	282.3	100	15
d18:0	302.3	284.3	90	25
S1P-d18:1	380.3	264.3	80	20
S1P-d18:0	382.3	284.3	95	10
AOD	314.3	269.3	80	25
AOD-C14:0	524.6	296.3	100	35
AOD-C14:1	522.6	296.3	100	35
AOD-C16:0	552.6	296.3	100	35
AOD-C16:1	550.6	296.3	100	35
AOD-C18:0	580.6	296.3	100	35
AOD-C18:1	578.6	296.3	100	35
AOD-C20:0	606.6	296.3	100	35
AOD-C20:1	604.6	296.3	100	35
AOD-C22:0	634.6	296.3	100	35
AOD-C22:1	632.6	296.3	100	35
AOD-C24:0	664.6	296.3	100	35
AOD-C24:1	662.6	296.3	100	35
AOD-C26:0	692.6	296.3	100	35
AOD-C26:1	690.6	296.3	100	35
Cer-d18:1/C12:0	482.5	264.3	100	35
SM-d18:1/C12:0	647.6	184.2	100	35
GluCer-d18:1/C12:0	644.7	264.3	70	40
DHCer-d18:0/C14:0	512.5	266.3	100	35
DHCer-d18:0/C16:0	540.5	266.3	100	35
DHCer-d18:0/C18:0	568.6	266.3	100	35

DHCer-d18:0/C20:0	596.6	266.3	100	35
DHCer-d18:0/C22:0	624.6	266.3	100	35
DHCer-d18:0/C24:0	652.7	266.3	100	35
DHCer-d18:0/C24:1	650.6	266.3	100	35
Cer-d18:1/C14:0	510.5	264.3	100	35
Cer-d18:1/C16:0	538.5	264.3	100	35
Cer-d18:1/C18:0	566.6	264.3	100	35
Cer-d18:1/C20:0	594.6	264.3	100	35
Cer-d18:1/C22:0	622.6	264.3	100	35
Cer-d18:1/C24:0	650.6	264.3	100	35
Cer-d18:1/C24:1	648.6	264.3	100	35
DHCer-m18:0/C14:0	496.5	268.3	120	50
DHCer-m18:0/C16:0	524.5	268.3	80	35
DHCer-m18:0/C18:0	552.6	268.3	80	30
DHCer-m18:0/C20:0	580.6	268.3	85	45
DHCer-m18:0/C22:0	608.6	268.3	80	35
DHCer-m18:0/C24:0	636.7	268.3	120	40
DHCer-m18:0/C24:1	634.7	268.3	85	35
Cer-m18:1/C14:0	494.5	266.3	110	35
Cer-m18:1/C16:0	522.5	266.3	120	40
Cer-m18:1/C18:0	550.6	266.3	125	40
Cer-m18:1/C20:0	578.6	266.3	125	35
Cer-m18:1/C22:0	606.6	266.3	105	40
Cer-m18:1/C24:0	634.7	266.3	80	55
Cer-m18:1/C24:1	632.7	266.3	110	35
SM-d18:1/C14:0	675.5	184.3	100	35
SM-d18:1/C16:0	703.6	184.3	100	35
SM-d18:1/C18:0	731.6	184.3	100	35
SM-d18:1/C20:0	759.6	184.3	100	35
SM-d18:1/C22:0	787.7	184.3	100	35
SM-d18:1/C24:1	813.7	184.3	100	35
SM-d18:1/C24:0	815.7	184.3	100	35
DHSM-d18:0/C14:0	677.6	184.3	100	35
DHSM-d18:0/C16:0	705.6	184.3	100	35
DHSM-d18:0/C18:0	733.6	184.3	100	35
DHSM-d18:0/C20:0	761.7	184.3	100	35
DHSM-d18:0/C22:0	789.7	184.3	100	35
DHSM-d18:0/C24:0	817.7	184.3	100	35
HexCer-d18:1/C14:0	672.5	264.3	70	40
HexCer-d18:1/C16:0	700.6	264.3	65	45
HexCer-d18:1/C18:0	728.6	264.3	60	45
HexCer-d18:1/C20:0	756.7	264.3	70	50
HexCer-d18:1/C22:0	784.7	264.3	70	50

HexCer-d18:1/C24:1	810.7	264.3	70	50
HexCer-d18:1/C24:0	812.8	264.3	110	60
HexDHCer-d18:0/C14:0	674.5	266.3	70	40
HexDHCer-d18:0/C16:0	702.6	266.3	65	45
HexDHCer-d18:0/C18:0	730.6	266.3	60	45
HexDHCer-d18:0/C20:0	758.7	266.3	70	50
HexDHCer-d18:0/C22:0	786.7	266.3	70	50
HexDHCer-d18:0/C24:1	812.7	266.3	70	50
HexDHCer-d18:0/C24:0	814.8	266.3	110	60

Supplementary Table S2. qPCR primer sequences used in this study

Gene	Forward primer Sequence (5'-3')	Reverse primer Sequence (5'-3')
SPTLC1	GCAGTGTTGAAGGAAAAGTGCGG	CAGTGCTCTCTTCCAGTTGTAGG
SPTLC2	CCAGACTGTCAGGAGCAACCAT	TTCGTGTCCGAGGCTGACCATA
SPTLC3	CCGACTCTCAGGTGCAACCATA	TGTAGACACCCTCCACCAGGAT
CERS1	GTCACCCTGCAACCGTGCCAC	AGGTCGAAGACGACTGTCCACT
CERS2	GCCTTGCTCTTCCTCATCGTTC	TGCTTGCCACTGGTCAGGTAGA
CERS3	CATGATCTTGCAGGTCCTTCACC	CTCGTCATCACTCCTCACATCC
CERS4	GACCTTCTCCTACAGTGCCAAC	GTCGCACACTTGCTGATACTGC
CERS5	GGTCACCATTGGGCTTATCTCC	GTGTCACAGAGCCGCTGATACT
CERS6	GACGCAATCAGGAGAAGCCAAG	GGTAGTTGTACCAGCAATGCCTC
DEGS1	CAGCTAGTCTGCAAGCCAC	CTCTGGATACTTTGCCAGGAT
FADS3	TGGGATCCCCAGGACTCG	GAAGGCATCCGTGGCGT
SPHK1	TCCCTGTAGGAAGAGTGGGT	GTGCCAGGACTAGCACAAAG
SPHK2	CACGTGCTTCCCATGATCTC	CGTGGTTCTGTCGTTCTGTC
SGPP1	CTGGTGTTCTCTAGTTTGCCTAAG	GGTTGAAGTTGTCAATCAGGTCC
SGMS1	CGAAAGCGTTCGCACCAG	GCTGTCGTCACGTTGCAC
SGMS2	GTGTGCTCCAAAGCTCAATGG	GTGTGACCGCTGAAGAGGAA
SMPD1	CCCTGGCTGCTCAGTTCTTT	GGTACACACGGTAACCAGGAT
UGCG	TGGCTATCATCTACACCCGA	ACCCCTTTCAGTGGTTTCAGA
GBA	CTTGCAGGGCTAACCTAGTG	GCTTGGGACATTCCTCTCTG
GBA2	CAGTCATCGCTGACCAATTCAC	CCTGGTAGTCATGGGGCAAG
SERPINE1	TGGTTCTGCCCAAGTTCTCC	CACCGTGCCACTCTCGTTC
HK1	GCCTATTACTTCACGGAGCTGA	TGAAATCTCCCTTTTCAGAGCCA
EGLN3	GCTTCTGCTACCTGGACAACT	AGCCACCATTGCCTTAGACC
18S rRNA	ACCCGTTGAACCCCATTCGTGA	GCCTCACTAAACCATCCAATCGG

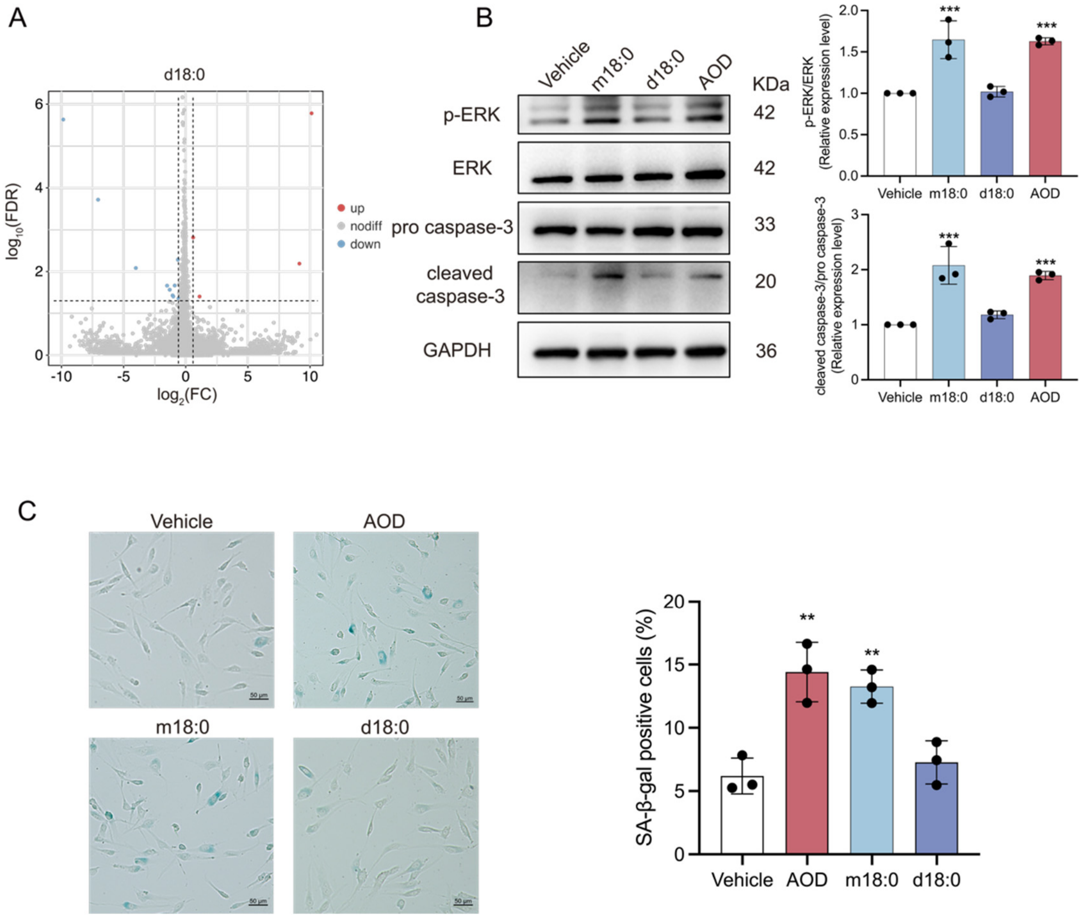

Figure S1. Volcano plot of differentially expressed genes (d18:0-treated vs. vehicle control) and effects of AOD and m18:0 on cellular senescence and stress response signaling

(**A**) Volcano plot of RNA-seq data from HepG2 cells treated with d18:0 for 24 hours. Genes with a fold change > 1.5 and an adjusted *p*-value < 0.05 are considered significantly differentially expressed. (**B**) Immunoblotting of p-ERK, ERK, pro-caspase-3, and cleaved caspase-3 in HepG2 cell treated with 5 μM AOD, m18:0, or d18:0 for 24 hours. (**C**) SA-β-gal staining of the HUVECs cell treated with 1 μM AOD, m18:0, or d18:0 for 72 hours. Data are mean ± SD and were analyzed by one-way ANOVA followed by Dunnett's multiple comparisons test (B, C). Significance levels are *$p < 0.05$, **$p < 0.01$, with a sample size of n = 3.

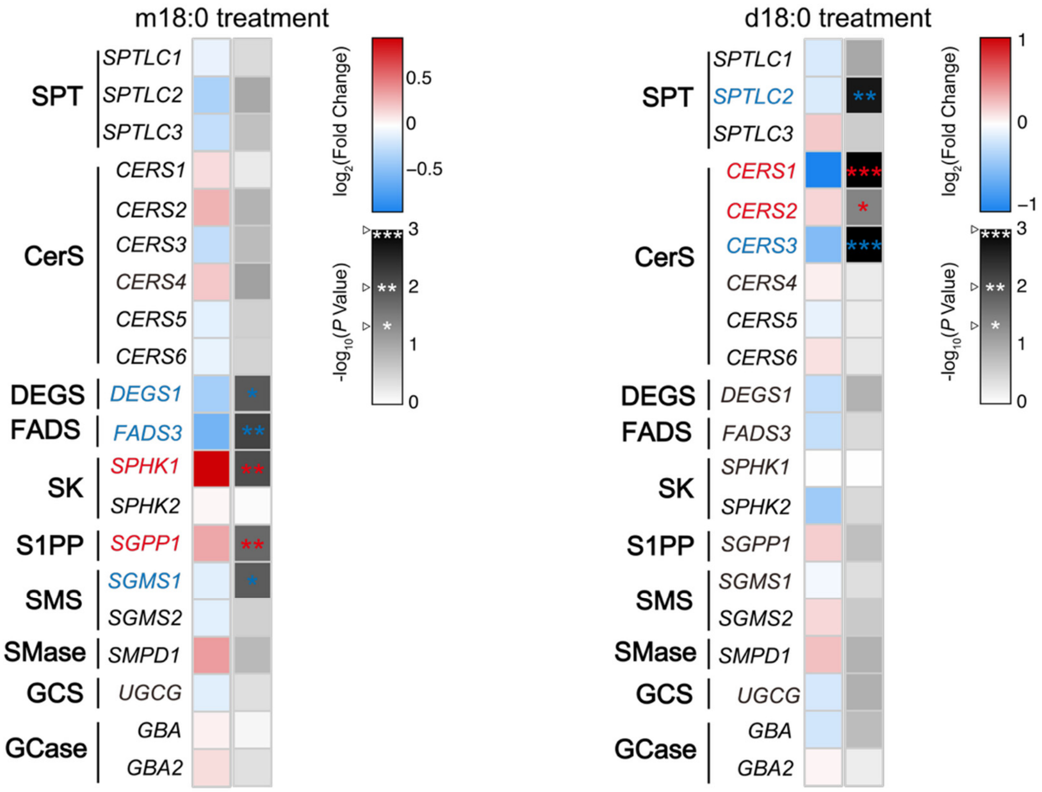

Figure S2. The regulation of sphingolipid metabolism-related genes by d18:0 and m18:0

Heatmap depicting mRNA expression levels of genes associated with the sphingolipid *de novo* synthesis pathway in HepG2 cells treated with m18:0 and d18:0 versus vehicle controls. The red-white-blue color gradient represents log_2-transformed fold changes (treated groups/vehicle groups) with red indicating upregulation and blue denoting downregulation. Corresponding $-log_{10}$(p-values) are encoded in a white-black gradient (low to high). Data were analyzed by two-tailed unpaired t-tests. Significance levels are $*p < 0.05$, $**p < 0.01$, $***p < 0.001$, with a sample size of n = 3.

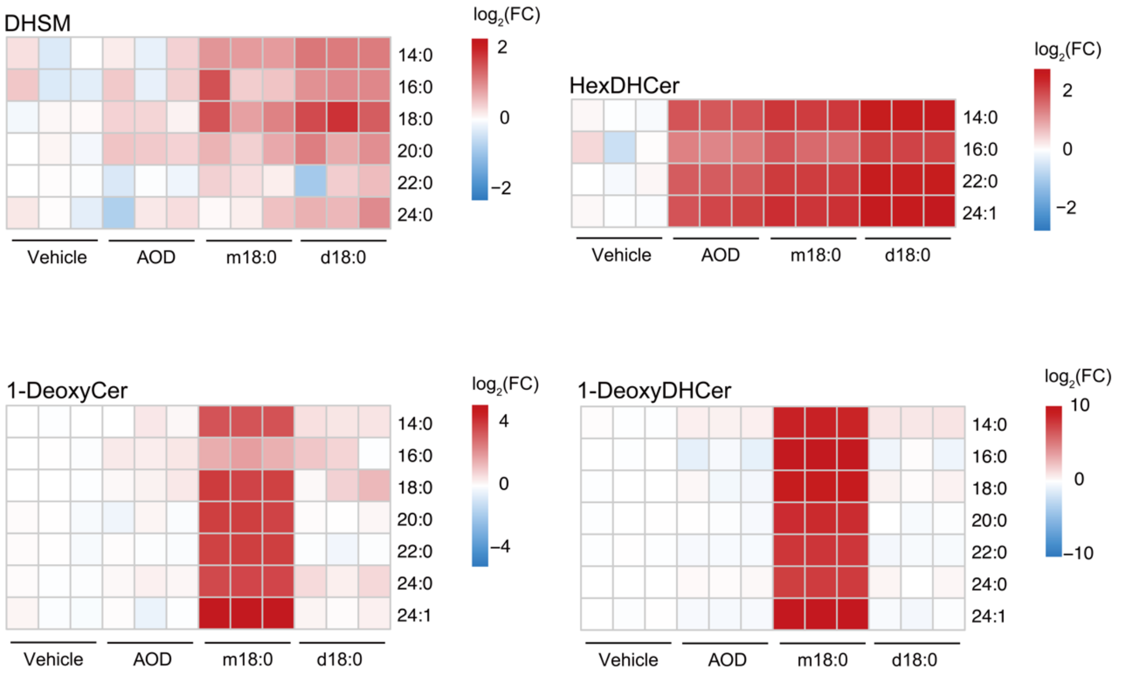

Figure S3. Targeted analysis of sphingolipids with differential *N*-acyl chain lengths by LC-MS/MS

Distribution of different acyl chain lengths of dihydrosphingomyelin (DHSM), dihydrohexosylceramide (HexDHCer), 1-deoxyceramide (1-DeoxyCer), and 1-deoxydihydroceramide (1-DeoxyDHCer) and in HepG2 cells after 24-hour treatment with 5 μM AOD, m18:0, or d18:0.
